# The significance of Emmanuel Levinas’ ethics of responsibility for medical judgment

**DOI:** 10.1007/s11019-022-10077-0

**Published:** 2022-07-31

**Authors:** Lazare Benaroyo

**Affiliations:** Interdisciplinary Centre for Research in Ethics, University of Lausanne, Anthropole Building, 1015 Lausanne, Switzerland

## Abstract

At a time when the practice of medicine is subject to technical and biopolitical imperatives that give rise to defensive bioethics, it is essential to revitalize the ethical dimensions of care at the very heart of the clinic, in order to give new meaning to the moral responsibility that inhabits it. This contribution seeks to meet this challenge by drawing on the ethical resources of the work of Emmanuel Levinas. In Levinas’ view, ethical responsibility is the response to the injunction, the interpellation, of the other’s face, and humaneness is conceived entangled in the other’s face. Against this background, I suggest that Levinas’ philosophical insight constitutes a turning point from a traditional to a new conception of responsibility that may bear great significance to a renewed understanding of an hermeneutics and an ethics of care. By drawing on this aspect of Levinas’ thought, I would like to reveal the ethical core of an ethics of clinical care that shapes a new insight on medical judgment.

## An introduction to clinical judgment

From an epistemological perspective, medicine may be considered as a practical science. Its aim is not primarily to know, but rather to act and intervene for the patient’s good. In this view, medicine is organized through actions that are carefully evaluated, planed, and rationaly driven by practical wisdom deriving from responsible choices while paying close attention to the real needs of patients.

As Georges Canguilhem states (Canguilhem 1994), feeling ill does not mean feeling abnormal in the sense of a deviation from the biological norm: for the patient, illness is characterized by a new pattern in his organism, a new form of adaptation to disruptions from the external environment, which translates into the development of a new vital norm. In this context, illness is a decrease in the ability to be normative: illness is not, in the eyes of the sick person, a loss of his normality but rather a reduction in his ability to establish new norms.

Hence, the patient’s quest for health can be interpreted as a search for the reestablishment of his organism’s autonomy – illness being experienced by the patient as the reduction in autonomy in the form of a decrease in the level of normative activity, or even a reduction of the whole organism to a unique norm.

Bearing in mind this phenomenology of the pathic experience, clinical practice thus transforms itself into a solicitude oriented towards a hermeneutic process, interpreting signs and symptoms towards a reconstruction of meaning, time, and the individual vital norm altered by illness. Accordingly, the healer should deliberate, taking incidental circumstances into account, in order to decide on the best possible good for each particular individual. The determination of this good rests on her ability to welcome the patient’s suffering and its meaning, and then make *choices* which guide her actions towards the patient’s recovery.

This approach enables an understanding of the individual as a being anchored in space and time, as demonstrated by his life’s narrative. Such an anamnesis can be of great help here: it enables, for example, engagement with the patient in a reconstructive task, through language, by examining the following themes:

- What is the patient’s lifestyle?
- What was the patient’s existence aimed at until now?
- What are the conflicts or tensions present in her existence?
- In what way does the patient involve her body in this confrontation?
- How does she adapt her time and vital space in relation to the illness?
- In what way does the patient put her existence at stake through her illness?
- What changes happened in her social network in relation to her illness?

These concerns for the patient as an individual can only draw the clinician’s attention towards the way in which illness affects her patient’s identity. The physician should explore the narrative registers through which suffering is expressed and especially to what makes the patient’s suffering particular, insights that emerge in the narrative.

Consequently, listening attentively to the patient’s narrative (i.e. to the way she refers to herself as the subject of her own story), and watching body language (during technical procedures for example), are elements enabling perception of how the patient’s identity has been affected by illness. The narrative registers convey how we perceive our belonging to a community, how suffering becomes inscribed in its own temporal evolution and alters the intimate perception of experienced time (Brody 2003). As Cheryl Mattingly notes (Mattingly 1994, p. 814):

Therapeutic success depends in part upon the therapist’s ability to set a story in motion which is meaningful to the patient as well as to herself. One could say that the therapist’s clinical task is to create a therapeutic plot which compels a patient to see therapy as integral to healing.

Drawing on this conception of clinical insight, rooted first in welcoming and then acting responsively, I propose to explore in this paper the many ethical faces of clinical care, and more particularly how Levinas’ thoughts may help us to grasp the ethical core of clinical judgment.

In order to better understand the significance of Levinas’ philosophy for an ethics of hospitality in the realm of clinical practice, it seemed to me worthwile to first recall Paul Ricoeur’s general understanding of medical judgment, which is today considered “of value for the further conceptual elaboration of the themes and insights of care ethics” (van Nistelrooij et al. 2014, pp. 486, Benaroyo 2011). This first step will help us to more precisely uncover the relevance of Levinas’ ethics of responsibility for medical judgment.

## Paul Ricoeur’s study of medical judgment

Paul Ricoeur explored medical practical wisdom in his study of prudential judgment first published in French in 1996 and translated into English in 2000 (Ricoeur 2000). Calling upon and adjusting the Aristotelian legacy, Ricoeur distinguishes three different and complementary stages in the elaboration of prudential judgment in the realm of clinical activity :

- First level *teleological judgment* that seeks to clarify in dialogue with the patient - in the realm of a *pact of care based on trust* - the ethical individual aim and the medical goal to be pursued, namely explore what suffering means in this singular case.
- The second level, called *deontological judgment*, performs the universalisation of the first order individual judgment described above in the realm of *a contract of care*, treating, among other things, conflicts within or outside the sphere of clinical intervention, as well as dealing with social and institutional ackowledged norms, such as informed consent, distributive justice and rules of health care allocation.
- The third level, is the *prudential judgment*, the moment of implementing *practical wisdom*, that reaches completion after a common – usually team-based deliberative process. It is the moment of the wise decision taking into account first and second order previous judgments and integrating them to formulate an *personalized decision*. In Ricoeur’s view, this decision draws its ethical roots out of the teleological level that gives meaning to the whole medical act, namely that orients the *telos* of the medical intervention.

This three-stage architecture of medical ethics, characterized by the integration through practical wisdom of three differents orders of ethical judgments, may be viewed, I would argue, as the paradigmatic basic structure of practical wisdom in clinical medicine.

Yet, Ricoeur’s architecture lies on the ethical proper dimensions of the clinical encounter, namely the teleological layer where the *telos* of the medical intervention can be defined with the patient. For Ricoeur, the ethical core of this encounter lies in a *pact of care based on trust*. This pact structures for him ethically the clinical act : on the one side the patient, with the desire to be relieved from the burden of suffering, and on the other side, the health caregiver who promises to help the suffering patient.

Now, based on my clinical experience, I think that this first level ethical judgment, as conceived by Ricoeur, is in some way questionable from a clinician’s perspective. It is precisely by critically reflecting on this level that I would like to raise a question to which Levinas’ conception of ethical responsibility may bear great significance. In my view, indeed, establishing a *pact of care based on trust* is precisely one of the most challenging issue of clinical practice : instead of being a starting point for care – as Ricoeur takes it for granted - I see it rather as an end point to be realized before being able to reach to the completion of pratical wisdom.

Hence, instead of relying on the assumption that a pact of care may be sealed by two autonomous partners, as Ricoeur suggests, clinical experience shows that the asymmetry of the clinical encounter may be interpreted first and foremost as a moral stance, a call for the physician’s “response-ability” – a response to the call of the patient’s vulnerability starting with the words : “Here I am to listen to the meaning of your suffering and help you to restore your wounded self”. This answer is, in my view, the basic ethical layer upon which trust may be built and constitutes the crucible of a pact of care.

My suggestion is then the following: it is precisely at this level that Levinas thought may shed light on the very nature of a caring attitude in a clinical world ; it is on this basis that practical wisdom may be implemented in a clinico-technical and social world according to Ricoeur’s view. This reverse understanding of the basic ethical foundation of ethical responsibility is precisely what Levinas’ philosophy can help us to comprehend and implement in clinical care. I would now like to explore this issue to attempt to ground an ethics of care rooted in welcoming and hospitality.

## Emmanuel Levinas’ ethical insight

It may seem at first glimpse a unfaithfulness to Levinas’ thought to call upon it in an attempt to build up a philosophy of clinical care. I am fully aware of this potential difficulty. What I would like to do in this paper is to explore this issue in light of Levinas’ ethical insight in order to approximate the ethical core of the face-to-face with the patient according to his approach of what he characterizes a human encounter. By drawing on this aspect of Levinas’ thought, I would like to get closer to the ethical core of clinical care.

Levinas’ philosophy^1^ starts by taking a critical distance from Heidegger. In distinguishing his own project from Heidegger’s, Levinas states that philosophy is more than the questioning of Being, but strives to move beyond the tension between being and a good beyond being – between ontology and ethics – which is constitutive of Levinas’ mature philosophy and guides the unfolding of the ethical problematics of his major work (Levinas 1985, pp. 37–44).

Through his reflection in his work on the absolute alterity of the other and on the ethical relationship as an infinite, irrecusable responsability, Levinas proposes a radical rethinking of the central categories of ethical life – self, other, subjectivity, autonomy, rationality, freedom, will, obligation – and the very meaning of the ethical (Levinas 1969).

For Levinas, ethics is not merely one branch of philosophy among others, secondary to the question of ontology, epistemology or theory of knowledge. It is not a superstructure grafted onto an antecedent relationship of cognition. Rather, Levinas maintains, ethics is “first philosophy”. As he himself makes clear, the aim of his work is not to construct an ethics, or a morality, in the sense of a system of rationaly justified precepts or norms capable of guiding human action and behavior ; rather, his work opens the question of the ethical as the “extreme exposure and sensibility of one subjectivity to another”.

The philosophical language of Levinas enacts a discourse in terms of “otherwise than being” (Levinas 1981) that frees the subjectivity from an ontological program. The individual is not just *Dasein*, he is also the site of transcendence, responding to the unfulfillable obligation towards the Other : *being-for-itself* is conditional to the unconditioned responsibility of *being-for-the-other*.

This moral endeavour, based on peace for the other, is an indication of the radical challenge to thought posed by the philosophy of Levinas. In our age, Levinas’ thought raises the question of the infinite demand of the ethical relation.

To sum up, for Levinas, the preconscious experienced responsibility for the other is the fundamental ethical layer of the responsible self. A responsibility that is a radical heteronomy – though not a principle of heteronomy –, an inescapable preconscious subjection to the other.

## The significance of Levinas’ ethics for medical judgment

To return to our inquiry centered on the philosophical basis of medical care, it is worth noting that this inescapable condition is, for Levinas, first of all reflected in the human sense of corporeity that is vulnerability. The incarnated subject, experienced as vulnerability - rather than as a detached game of consciousness - is, for Levinas, the place of the call for the other, an inescapable call, before any choice, before the birth of liberty. Embodiment as vulnerability is the cornerstone of Levinas’ moral approach : the body – whether healthy or ill - experienced as vulnerability, is the locus of my exposure to the others - of a call for responsibility. Again, not a responsibility assumed by a conscious decision, but an inescapable call, not a free choice, but the ethical condition of my own self.

For Levinas, this responsibility is revealed by the other’s face. The face of the other reminds us that the ethical meaning of an encounter is not totally contained within the limits of consciousness or within the social superstructures of a world of moral strangers.

Now, what do Levinas’ foundations of an ethics of response mean in the realm of medical care ?

In Levinas’ view, facing suffering reveals my own humanity, within a space of shared vulnerability, of “familiar” fragility. It is on the basis of this Levinasian approach to suffering - conceived as a dialectic between the interpellation of the patient and the availability of the carer - that a climate of trust can blossom, anchored in a common ground of humanity. My ethical responsibility as a caregiver thus includes both a response to the interpellation elicited by the call of the other and a movement of self-reflection, a source of acceptance of the suffering other. The true “visitation” of the patient - beyond the clinically codified visit - implies accepting his radical otherness, welcoming him into myself, in a movement of hospitality that mobilizes my own vulnerability. It is at the heart of this dialogue between two vulnerabilities that trust can be born. An ethical approach such as Levinas conceives it, aims above all at making the humanity of the other man come about at the same time as my own humanity.

Thus, the other’s vulnerability, particularly in the case of illness, is, for Levinas, the very incarnated locus of ethics, eliciting one’s own vulneralibity and therefore responsibility-for-the-other human being:

Is not the evil of suffering – extreme passivity, helplessness, abandonment and solitude – also the unassumable, whence the possibility of a half opening, and, more precisely, the half opening that a moan, a cry, a groan or a sight slips through – the original call for aid, for curative help, help from the other me whose alterity, whose exteriority promises salvation ? Original opening toward merciful care, the point at which (…) the anthropological category of the medical, a category that is primordial, irreducible and ethical, imposes itself. For pure suffering, which is intrinsiquely senseless and condemned to itself with no way out, a beyond appears in the form of the interhuman. (Levinas 1998, pp. 93–94)

What does this way of conceiving responsibility-for-the-other mean in the realm of medical practice ?

In clinical medicine, the unfolding of the responsibility-for-the-other entails being aware of the radical otherness of the other, as well as at the same time being aware of the common vulnerability that links physician and patient, namely to the inescapable responsibility one owes to the other, more particularly for the physician, to the responsibility to care for the other in his radical otherness.

As Levinas points out, the faithfulness of this responsible posture requires a personal availability (“disponibilité” in Gabriel Marcel’s vocabulary) to be open to welcome the otherness of the other. In Levinas’ thought, availability is the correlative central issue to ethical reponsibility-for-the-other, it is the support on which the unfolding of this responsibility rests. Availability means the ability to open up in oneself a place for the other: namely create a space for otherness in oneself. Ethics *as* hospitality in Jacques Derrida’s words, is, I think, the most appropriate way to qualify the ethical core of clinical care in the wake of Levinas’ conception of responsibility.

In the clinical realm, this means that the health care provider should be able to listen to the otherness *first in himself* before opening himself to the radical otherness of the patient. This understanding of *hospitality* may pave the way for a trustful caring environment: the healer’s welcoming of the other can express itself and be perceived by the patient as a sign of a common and shared meaning – of ethical community - that is not a response but a question, a question which is always open.

Accordingly, the Levinassian understanding of common humanity may be grasped as the inescapable condition of elaborationg a pact of care based on trust. This pact may be conceived, in this view, as a second order ethical dimension of clinical activity. This dimension rests on a preconscious Levinassian first order ethical awareness to the human breadth of responsibility–for-the-suffering-other, meaning that the face of the suffering other always inescapably calls upon a welcome.

And to go a step further, I could argue with Jacques Derrida :

[The health care giver] must first think the possibility of the welcome in order to think the face and everything that opens up or is displayed with it : ethics, metaphysics or first philosophy, in the sense that Levinas gives to these words. The welcome determines the “receiving”, the receptivity of receiving as the ethical relation. (Derrida 1999, pp. 25)

In other words, ethical discourse starts with the interruption of the self by the self. “It is I who support the Other and am responsible for him. […] My responsibility is untransferable, no one could replace me. In fact, it is a matter of saying the very identity of the human I starting from responsibility, that is, starting from this position or deposition of the sovereign I in self consciousness, a deposition which is precisely its responsibility for the Other.”(Levinas 1985, pp. 100–101).

In this sense, one might call this moral stance, ethics *as* hospitality (Benaroyo 2021).

## Conclusion and perspective

Hence, in the realm of clinical medicine we are now facing the *paradox* that lies at the root of the unfolding of ethical responsibility in the sense that Levinas gives to this word. The responsible healer has a double face of Janus: on the one side, the naturalistic rational attitude, to recall Husserl’s terms, is necessary to observe the patient with objectivity in order to master his disease processes, and on the other side, the personal pre-rational attitude entails hospitality to welcome the other’s vulnerability as an ethical calling. It is out of a synthesis of both opposite attitudes – which respectively call on radically different goals – that responsibility-for-the-other, guided by practical wisdom in the vein of Ricoeur’s thought, may be effected in clinical care.

In my view, this is the inevitable ethical challenge awakened by Levinas’ ethics, that every clinician should face and to which she should be prepared by entering clinical practice. My vision of an ethical medicine hinges then on the necessary complementarity of both sides of the Janus face: I would suggest that both sides may take one melded with the other with the following ethical steps, paving the way for a renewed conception of practical wisdom cautiously articulating Ricoeur’s philosophy of action and Levinas’ “ethics of responsibility” all over the irreducible moments of its unfolding:

- First, an ethical awakening to the vulnerability of the suffering other in his radical otherness – ethics *as* hospitality and love.
- Second, on the basis of the first step, elaborating a pact of care based on trust - ethics *as* justice and care.
- Third, performing the technical step leading to healing and curing – ethics *as* moral norms regulating applied science.
- Fourth, reaching to completion the prudential judgment drawing on practical wisdom that chooses and decides what is the right individualized care – ethics *as* practical wisdom.

Thus, attention to the different moments in the clinical judgment we have highlighted appears to mark the path of an ethics of responsibility – drawing its sources from an ethics of hospitality and responsiveness – which revitalizes the links between ethics and clinical practice (Benaroyo 2015). The figures of care which outline these different moments thus become living metaphors attesting to the intimate ties between ethics and medicine.

## Footnotes

1 Emmanuel Levinas was born in Kovno, Lithuania in 1906, to Jewish parents. In 1923, he went to Strasbourg (the closest French city to Lithuania) in order to study philosophy under such teachers as Charles Blondel and Maurice Pradines. In 1928—29, he attended a series of lectures given in Freiburg by Husserl on phenomenological psychology and the constitution of intersubjectivity. It was at this time that he began to write his dissertation on Husserl’s theory of intuition. He also discovered Heidegger’s *Being and Time.* His book *De l’existence à l’existant*, published in 1947 described his anonymous existence, and the bouts of insomnia, sleep, horror, vertigo, appetite, fatigue and indolence - that he had endured in captivity. This book attracted little attention, although Jean Wahl invited him in the same year to give a course for his students, the text of wich was published soon after under the title *Le temps et l’autre*. This situation changed in 1961, however, when Levinas published *Totalité et infini. Essai sur l’extériorité*. Suddenly a master was revealed, one not only capable of renewing twentieth century phenomenology but one who also combined a radical critique of Western philosophy. His second major book *Autrement qu’être**ou au-delà de l’essence*, which appeared in 1974, represents a new stage in his thinking, one even more original than the former. The philosophical work of Levinas became known in America through the early translation of his main texts, *Totality and Infinity**:**An Essay on Exteriority* in 1969 and *Otherwise than Being**or Beyond Essence* in 1981.

## References

[CR1] Benaroyo, L. 2011. ‘Constructing medical wisdom: The significance of healing narratives for clinical practice’. In *Sprache und Wissenserwerb. Ein interdisziplinärer und interkultureller Zugang/Language and Acquisition of Knowledge. An Interdisciplinary and Intercultural Approach, *eds. A. Neschke and H. R. Sepp, 233–244. Nordhausen: Verlag Traugott Bautz GmbH.

[CR2] Benaroyo L, ten Have H (2015). “Hermeneutics”. Encyclopedia of Global Bioethics.

[CR3] Benaroyo L (2021). Soin et bioéthique. Réinventer la clinique.

[CR4] Brody, H. 2003. *Sories of Sickness.* Second Edition. Oxford: Oxford University Press.

[CR5] Canguilhem G, Delaporte Fr. (1994). The Normal and the Pathological. A Vital Rationalist. Selected Writings from Georges Canguilhem.

[CR6] Derrida J (1999). Adieu to Emmanuel Levinas.

[CR7] Levinas E (1969). Totality and Infinity: an Essay on Exteriority.

[CR8] Levinas E (1981). Otherwise than Being or Beyond Essence.

[CR9] Levinas E (1982). Ethics and Infinity. Conversations with Philippe Nemo.

[CR10] Levinas E (1998). Useless suffering in *Entre Nous*. On Thinking of the Other.

[CR11] Mattingly Ch (1994). The Concept of Therapeutic « Emplotment ». Social Science and Medicine.

[CR12] van Nistelrooij I, Schaafsma P, Tronto JC (2014). Ricoeur and the ethics of care. Med Health Care and Philos.

[CR13] Ricoeur, P. 2000. Prudential Judgment, Deontological Judgment and Reflexive Judgment in Medical Ethics. In *Bioethics and Biolaw. Judgment of Life*, eds. P. Kemp, J. Rendtorff, and N. Mattsson Johansen, vol. 1, 15—26. Copenhagen: Rhodos International Science and Art Publishers.

